# Building a Circular Economy for Lithium: Addressing Global Challenges

**DOI:** 10.1002/gch2.202400250

**Published:** 2024-11-12

**Authors:** Alessandra Zanoletti, Bianca Maria Bresolin, Elza Bontempi

**Affiliations:** ^1^ INSTM and Chemistry for Technologies Laboratory University of Brescia Via Branze 38 Brescia 25123 Italy

**Keywords:** critical raw materials, critical raw materials act, green transition, lithium‐ion battery, mining, recycle, sustainability

## Abstract

As countries worldwide race toward a green transition, the demand for electric vehicles is surging, and with it comes a growing need for batteries. However, the push for increased domestic mining to secure these materials raises significant concerns about environmental sustainability. Even with stringent regulations, the environmental impact of mining can be profound, posing risks such as biodiversity loss, water pollution, and broader ecological damage. Furthermore, geopolitical tensions could arise as countries whose economic interests are threatened by these initiatives may react adversely. Local communities might also resist mining projects due to concerns over environmental degradation, health risks, and disruptions to their livelihoods. Given the critical importance of metals in the ecological transition, this challenge must be approached with the same urgency and global coordination as a pandemic response. Just as the world mobilized unprecedented resources to tackle COVID‐19, a similarly robust approach is necessary to ensure the availability of critical metals for a sustainable future. This paper suggests potential pathways for academic, technological, and societal advancements within the framework of a circular economy for lithium, aiming to secure a sustainable supply of this essential resource.

## Graphical abstract

The paper discusses the implications of the growing demand for lithium due to the global shift toward electric vehicles and renewable energy. It highlights the environmental and geopolitical challenges associated with increased lithium mining. The paper also emphasizes the need for sustainable practices, recycling, and global cooperation to ensure a steady supply of this critical resource while minimizing ecological impact.

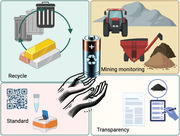

## 1 Introduction

The energy transition policies have significantly amplified the request for some raw materials, as the new energy technologies rely heavily on critical metals to meet the Paris Agreement goal of limiting global temperature rise to below 2 °C. Among these materials, lithium stands out due to its crucial role in some energy technologies, particularly as a key element in batteries for electricity grids and electric vehicles, thanks to its unique chemical‐physical characteristics.^[^ ^1^ ^]^

However, the availability of critical raw materials (CRMs) is increasingly at risk, influenced by a range of factors including the concentration of production, economic and political constraints on expanding production capacities, and widespread export restrictions. The European Union's Critical Raw Materials Act (CRMA) has emerged as a strategic initiative aimed at securing the supply of these essential materials, which are vital for both green and digital transitions.^[^ ^2^ ^]^ As global demand for CRM grows, the CRMA advocates for bolstering domestic mining and processing capacities within the EU, to reduce dependency on external suppliers, especially from geopolitically sensitive regions like China. This initiative is presented as a proactive measure to enhance the EU's strategic autonomy, stabilize supply chains, and mitigate vulnerabilities to global market fluctuations and political tensions.

Nevertheless, the push for increased domestic mining has raised concerns regarding environmental sustainability. Critics argue that such a move could result in significant environmental degradation,^[^ ^3^ ^]^ potentially compromising the EU's leadership in global sustainability. Even with stringent regulations, the environmental impact of mining is substantial, posing risks such as biodiversity loss, water pollution, and ecological damage. Moreover, the focus on mining could divert resources and attention from innovation in alternative materials, possibly slowing the development of more sustainable or less resource‐intensive technologies. There is also the potential for geopolitical tensions, as countries whose economic interests are threatened may react negatively. Additionally, local communities might oppose mining projects due to concerns about environmental degradation, health risks, and disruptions to their livelihoods.^[^ ^4^ ^]^ For example, in Chile lithium extraction practices have significantly altered the hydrological landscape, impacting indigenous communities reliant on water resources for agriculture and daily life. These processes also generated environmental and social challenges under the guise of “green energy” solutions. This situation underscores the paradox in lithium mining, where the global North's green mobility efforts, aimed at reducing carbon emissions, impose extractive pressures on the Global South, thus perpetuating a cycle of socio‐environmental injustice.^[^ ^5^ ^]^ Communities in the Atacama region have increasingly called for water justice and a reassessment of policies that prioritize material demand over local ecosystems and Indigenous rights.^[^ ^6^, ^7^ ^]^

While the CRMA presents an opportunity for the EU to position itself as a global leader in the ethical sourcing of raw materials, potentially setting international standards and encouraging other regions to adopt more sustainable practices, this leadership could be compromised if the environmental and social costs of domestic mining are not adequately addressed, leading to criticism from environmentalists and human rights advocates.^[^ ^4^ ^]^

This paper delves into the current state of lithium availability and offers insights into its future, drawing on the latest developments in the field. It aims to inform non‐specialist readers, spark debate on controversial issues within the research community, and present a viewpoint that integrates economic, political, and scientific perspectives, particularly from the standpoint of material science. Given the critical importance of metals in the ecological transition, this challenge must be approached with the same urgency and global coordination as a pandemic response. Just as the world mobilized unprecedented resources to tackle COVID‐19, a similarly robust approach is necessary to ensure the availability of critical metals for a sustainable future. This paper suggests potential pathways for academic, technological, and societal advancements within the framework of a circular economy for lithium, aiming to secure a sustainable supply of this essential resource.

## 2 The Lithium Availability Panorama

Lithium‐ion battery (LIBs) cells consist of several key components, each essential for the battery's performance, stability, and energy storage capacity. The primary components include the cathode, anode, electrolyte, separator, and current collectors.^[^ ^8^ ^]^ The cathode is the positive electrode and typically contains lithium metal oxides such as lithium cobalt oxide (LiCoO₂), lithium manganese oxide (LiMn₂O₄), lithium nickel cobalt manganese oxide (LiNiMnCoO₂), or lithium iron phosphate (LiFePO₄). The choice of cathode material impacts the battery's energy density, voltage, cycle life, and safety. High nickel‐content cathodes, for example, provide higher energy density but may have stability and safety trade‐offs. The anode is the negative electrode, often made from graphite, which stores lithium ions during charging and releases them during discharging. The electrolyte typically is a lithium salt dissolved in a solvent mixture, generally a combination of organic solvents such as ethylene carbonate and dimethyl carbonate. This liquid facilitates the movement of lithium ions between the cathode and anode. Solid and gel polymer electrolytes are being researched to improve battery safety and reduce leakage risks. The separator is a porous membrane, usually made of polypropylene or polyethylene, that physically separates the cathode from the anode to prevent short‐circuiting. It allows lithium ions to pass through while blocking electrons, which is essential for the safe operation of the battery. Current collectors are thin metal foils, typically aluminum for the cathode and copper for the anode, that conduct electricity between the electrodes and the external circuit. They serve as paths for the flow of electrons during charge and discharge cycles.^[^ ^9^ ^]^

**Figure 1:**
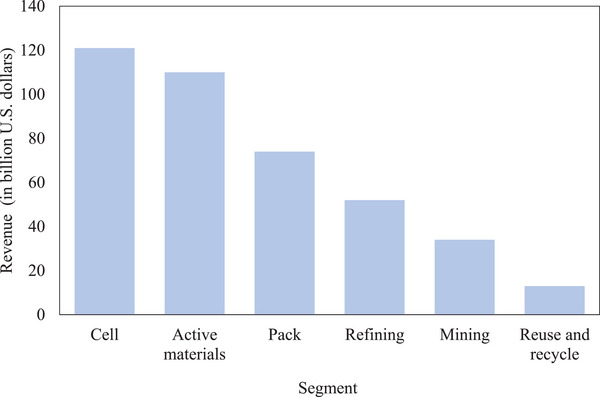
Expected revenue in billion U.S. dollars per segment of LIBs, e.g. cell, active materials, and pack, and per battery processing, e.g., refining, mining, and reuse and recycle.

The global lithium‐ion battery market is projected to generate revenues of approximately 400 billion U.S. dollars by 2030.^[^ ^10^ ^]^ However, the limited diffusion of new recycling technologies means that only about one‐third of this revenue—around 34 billion U.S. dollars—will be generated through the recycling of LIB minerals (see **Figure** **1**).

The rest will come from traditional mining activities, which have already seen significant growth, with global lithium production reaching a new high of 180 000 metric tons in 2023, up from just 28 100 metric tons in 2010.^[^ ^11^ ^]^ In the same year, lithium exploration reached an investment of $830 million, with a record of 77% growth, becoming one of the most explored commodities overall.^[^ ^12^ ^]^

By 2030, China is reported to dominate the industry of LIBs cell manufacturing, with revenue opportunities in this sector predicted to be significant. The revenue opportunities in the United States and Europe may reach levels comparable to those in China, but only if combined.^[^ ^10^ ^]^

Despite this potential, the recycling of lithium from waste batteries, which contain about 3–6% lithium by mass, remains a critical challenge. The elemental diversity and complex composition of batteries necessitate energy‐intensive processes like solvent extraction and high‐temperature treatments to selectively recover lithium.^[^ ^10^ ^]^

## 3 An Overview of Lithium Mining Technologies

Spodumene mining is a central method of lithium extraction, leveraging the mineral's high lithium content, with pure spodumene containing about 8 wt% lithium oxide (Li₂O). The process begins with the exploration to identify viable geological deposits. Then, the infrastructure is developed, complying with environmental standards to support mining operations.^[^ ^13^ ^]^ The extracted ore undergoes several stages of processing. Initially, mechanical treatments are used for beneficiation, where techniques like dense media separation, froth flotation, and magnetic separation isolate lithium‐rich spodumene from impurities.^[^ ^13^ ^]^ A crucial stage, roasting, involves heating spodumene at high temperatures to convert it from the α‐spodumene phase to the more valuable β‐spodumene form. Following roasting, the material undergoes acid treatment with sulfuric acid to further purify the lithium, removing remaining impurities. The resulting lithium is then precipitated, typically as lithium carbonate or lithium hydroxide, and refined to meet purity standards for battery production and other industrial applications.^[^ ^14^ ^]^

While lithium is essential to produce batteries used in electric vehicles and other clean energy technologies, its extraction from conventional sources, such as hard rock mines, has a substantial environmental impact. Moreover, the production of one metric ton of lithium generates around 15 000 kg of carbon dioxide and requires approximately 170 cubic meters of water, leading to significant landscape degradation.

Salt lake brines are another significant lithium source. Brine extraction involves initial pre‐treatment to remove minerals such as calcium and magnesium through chemical precipitation and filtration.^[^ ^13^ ^]^ The pre‐treated brine is then transferred to evaporation ponds, where solar energy promotes water evaporation, concentrating the lithium and other salts. When the brine reaches the desired concentration, lithium carbonate or lithium chloride is precipitated with specific chemicals, typically in dedicated ponds or reactors, and harvested once the purity requirements are met.^[^ ^15^ ^]^ Moreover, the extraction of brine and resulting changes in the salinity of nearby water bodies can lead to significant hydrological alterations, disrupting ecosystems and threatening the habitats of local wildlife.^[^ ^13^ ^]^

Alternative sources with respect to mineral extraction, like geothermal brine can offer a much lower environmental footprint, yet they still present challenges, particularly in terms of water usage and landscape impact.^[^ ^16^ ^]^ In recent years, direct lithium extraction (DLE) has emerged as an innovative approach to streamline lithium extraction from brines, avoiding the extensive time and land use required by evaporation ponds. DLE methods are generally categorized into three main types: adsorption, ion exchange, and membrane‐based processes, each focusing on selectively isolating lithium ions with increased efficiency and sustainability. These technologies capture lithium ions directly from brine solutions, significantly reducing the environmental impact associated with traditional methods.^[^ ^17^ ^]^ As DLE technology evolves, it holds promise for making lithium extraction more rapid, efficient, and environmentally sustainable.

Despite the potential of emerging extraction methods, such as DLE technologies^[^ ^18^ ^]^ and geothermal brine recovery, significant challenges persist. Key issues, including water consumption, chemical waste management, habitat destruction, and the depletion of natural resources, remain unresolved. Addressing these concerns is crucial to developing a more sustainable and efficient lithium supply chain.^[^ ^13^ ^]^

## 4 Limitations to the EU Policies’ Application

Recycling technologies for LIBs are crucial for recovering valuable materials, reducing environmental impact, and addressing resource scarcity. The main recycling methods include pyrometallurgical and hydrometallurgical processes.^[^ ^8^ ^]^ Pyrometallurgy involves smelting batteries at high temperatures to recover metals such as cobalt, nickel, and copper. The process burns off organic materials, including the electrolyte and plastic components, yielding a metal‐rich alloy that can be further refined. While effective, it is energy‐intensive and typically results in lower lithium recovery, as lithium tends to end up in slag or other by‐products. Recently, new efficient technologies based on microwaves have been also proposed.^[^ ^19^ ^]^

Hydrometallurgy is based on chemical leaching, using acids or other solvents to dissolve battery materials, for selective recovery of metals like lithium, cobalt, and nickel. Hydrometallurgical methods are generally more efficient at recovering lithium than pyrometallurgical ones and operate at lower temperatures. However, they require careful handling of chemicals and generate liquid waste.^[^ ^20^ ^]^

China dominated the global landscape in LIBs recycling capacity in 2021, boasting an impressive 188 000 tons of existing and planned recycling capacity annually.^[^ ^21^ ^]^

This placed China well ahead of Germany and the United States, which, despite their efforts, significantly lagged in this area. To further bolster its recycling capabilities, China has already implemented policies requiring designs that facilitate easier disassembly and promote domestic recycling.^[^ ^22^ ^]^

Since 2021, there have been additional initiatives aimed at expanding LIB recycling plants. The Fraunhofer Institute for Systems and Innovation Research^[^ ^23^ ^]^ has noted that while the majority of recycling capacity remains concentrated in East Asia, Europe is quickly scaling up its efforts. Europe could potentially boost its recycling capacity to an estimated 400 000 tons per year by 2025.

Under the framework of the CRMA, the European Union has introduced new regulations designed to strengthen the internal market for batteries and ensure fairer competition. These regulations set ambitious targets for producers, including the collection of 63% of waste portable batteries by the end of 2027 and 73% by the end of 2030.

**Figure 2:**
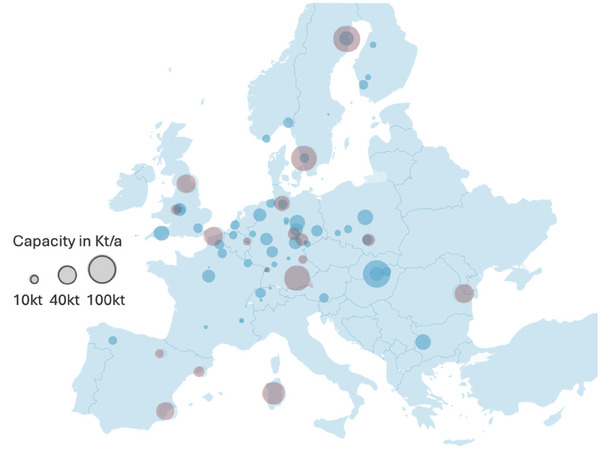
Distribution of current and planned LIBs recycling facilities across Europe, as projected by the end of 2024 (red bubbles). The size of each circle corresponds to the capacity of the facility, measured in tons per annum (t/a), with blue circles representing existing. Data from.^[^ ^23^ ^]^

Despite these proactive steps, several challenges persist. Although the production of new batteries is occurring at an unprecedented pace, the recycling market for LIBs is expanding more slowly. The map in **Figure** **2** illustrates the current and planned recycling facilities for LIBs across Europe as of June 2024. The blue circles denote facilities expected to be operational by the end of 2024, while the red circles represent announced projects. The high concentration of recycling sites in Central Europe is particularly striking, often reflecting the proximity to battery material producers, battery cell manufacturers, or automotive industries.

Despite significant efforts to enhance recycling capabilities, the reality is that many of the expected recycling facilities are not yet operational, leading to a continued and even increased reliance on mining activities. This trend is particularly concerning in areas already burdened with environmental and social issues. For example, the EU and Ukraine have signed a Strategic Partnership on Raw Materials and Batteries, which underscores Ukraine's substantial—though not fully quantified—reserves of critical raw materials, including lithium.^[^ ^24^ ^]^ Ukraine's reserves are considered vital for Europe's supply chain, as the country holds approximately 20 out of the 30 materials identified as critical by the European Union, such as titanium, lithium, rare earth elements, and manganese. However, this partnership raises concerns about the EU's ideological support for Ukraine, given the potential environmental and social ramifications.^[^ ^25^ ^]^ Moreover, similar agreements, such as the memorandums of understanding on critical raw materials recently signed between the EU and Serbia, have also sparked discontent. These developments highlight the ongoing challenges and the potentially problematic nature of the EU's reliance on external mining activities to meet its raw material needs, especially considering the slow progress in establishing adequate recycling infrastructure.

The ongoing reduction of ore grades generally leads to increased energy consumption, although technological improvements may help mitigate these higher costs. Given that most of the energy used in mining and refining is still fossil‐fuel‐based, emissions from the mining sector are expected to rise as demand increases and ore grades.^[^ ^26^ ^]^ The energy required for mining escalates exponentially with lower ore grades, potentially making further exploitation of these deposits economically unfeasible at some point.^[^ ^27^ ^]^ The mining industry is currently responsible for a share of global final energy consumption of approximately 17%.^[^ ^28^ ^]^ However, this share is projected to increase significantly in the future if current trends continue—marked by high economic growth coupled with material‐GDP coupling—potentially reaching 4–12% of the forecasted global final energy consumption.

**Figure 3:**
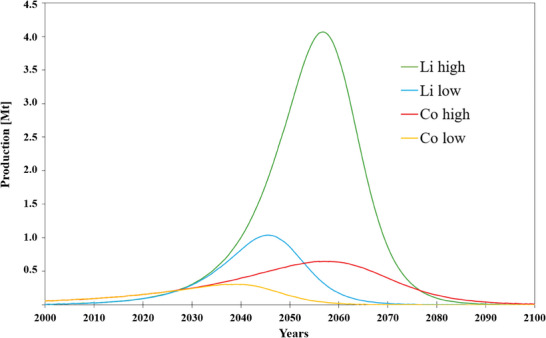
High and low scenario material production projection for Co and Li.^[^ ^29^ ^]^ The high and low scenarios serve to provide a range for future material availability under different economic and technological assumptions. These scenarios illustrate the pressing need for sustainable practices and innovations to extend resource lifespans, especially as global demand for materials like Li and Co intensifies in renewable and e‐mobility applications. Post‐2050, production is expected to decline in both scenarios due to the depletion of high‐grade ores and the diminishing availability of economically viable reserves. As accessible deposits are exhausted, reliance on lower‐grade ores will drive up extraction costs and environmental impacts, making sustained production economically difficult.

The cumulative production curves for Li and Co are reported in **Figure** **3**.^[^ ^29^ ^]^ They show the evaluated production trends, peaks of production, and depletion time for these strategic materials depending on the materials reserves and possible resources.

Ore deposits, or reserves, refer to natural accumulations of materials that are both technologically extractable and economically viable. Conversely, resources include deposits that may not currently be economically viable but could become so with advancements in extraction technologies or changes in economic conditions as demand for the material increases. However, it's crucial to note that not all resources may be accessible due to factors such as location.

Figure 3 also presents the estimated peak and depletion years for lithium and cobalt. The maximum theoretical peak of extraction is achieved once the highest quality resources have been exhausted, leading to a subsequent decline in extraction as poorer quality or lower concentration deposits are exploited. The curves shown were developed by analyzing historical annual production data and projecting these trends into the future, utilizing data on material reserves and resources. The projections consider both Low and High production scenarios, depending on varying levels of material demand.

The high and low production scenarios are based on varying assumptions regarding available reserves, resources, and depletion rates, which influence the projected production peak and subsequent decline.

The low scenario primarily relies on known reserves, which are the economically viable portions of resources. This scenario assumes a high depletion rate, meaning the material will be exhausted faster due to higher extraction intensity.

The high scenario includes broader resources—the materials that may not currently be economically feasible to extract but could become accessible with technological advancements and favorable economic conditions. The depletion rate is moderate here, as this scenario assumes sustainable extraction over a longer period.

It is important to emphasize that the high production scenario is considered improbable. Moreover, the existence of inaccessible reserves could increase the potential for conflicts, adding a layer of complexity to future resource management. After 2050, production declines in both scenarios are anticipated due to the depletion of high‐grade ores and reduced availability of economically viable reserves. As easily accessible deposits are exhausted, reliance shifts to lower‐grade ores, increasing extraction costs and environmental impact, making continued production economically challenging.

The clean energy transition, which aims to drastically reduce greenhouse gas emissions, might encounter a rebound effect if the implications of increased mining activity are not seriously considered. By fragmenting nature into resources solely for consumption, we overlook the limits that could lead to the collapse of Earth's ecosystems.^[^ ^30^ ^]^

Additionally, the focus on mining might divert resources and attention away from innovation in alternative materials, potentially hindering the development of more sustainable or less resource‐intensive technologies.

## 5 Future Actions

Transitioning away from fossil fuels inevitably leads to a reliance on materials, many of which come with significant supply risks. Energy production is impossible without these materials, making it crucial to ensure a sustainable supply chain. This requires a deep understanding of the complex interdependence between energy, materials, and the environment. The challenges involved are multi‐dimensional and intricate, particularly when considering the serious social issues that mining activities can bring to the forefront.

The mineral wealth that is the natural heritage of both current and future generations must be valued, considering not only the immediate costs of extraction but also the long‐term impacts on future generations when these deposits are eventually depleted. This approach is essential to fostering a true sense of conservation.

Apart from some evident considerations, concerning for example the mining practices, that must be both environmentally and socially sustainable, some other urgent actions must be considered. Some actions that were put in place to manage the pandemic should be reconsidered and adapted for the present need to reach a sustainable energy transition.

### 5.1 Take a Global Accounting for Mining Monitoring

Mining operations have historically resulted in significant amounts of valuable metals ending up in tailings, primarily due to their low concentrations. Decades, or even centuries ago, the extraction of these metals was often deemed unprofitable, lacked practical applications, or was hindered by the absence of adequate recovery technologies. Today, however, many mining companies are re‐examining these tailings to determine the feasibility of extracting the valuable metals that have since accumulated. For example, previous methods for processing materials containing rare earth elements were inefficient, causing large amounts of these crucial elements to be discarded as waste.^[^ ^30^ ^]^

Despite our resistance to the environmental and social impacts of mining activities occurring close to home, we often push these extractive operations to other countries, where environmental and social standards may be lower or non‐existent. Nevertheless, we continue to demand the constant innovation of technological products, which heavily rely on mining. As governments seek to reduce reliance on foreign raw materials, there is increasing pressure to reinvigorate domestic mining—a strategy that is likely to encounter social resistance.

The challenge of raw material extraction is a global responsibility. It is imperative that all nations systematically account for the extraction and depletion of these resources. A comprehensive global framework for monitoring the ongoing depletion and environmental impact of mining should place the intrinsic value of nature on an equal footing with economic considerations.

### 5.2 Recycling and Recovery Technologies

The collection and proper recycling of electronic waste are currently inadequate, creating a significant bottleneck in the recycling process. Only 17.4% of the electronic waste generated globally is documented as being collected and recycled.^[^ ^8^ ^]^ This is in accord with the limited availability of recycling facilities (Figure 2).

There are a lot of new emerging technologies for lithium recovery from LIBs. Recently, much attention has been paid to pyro‐hydrometallurgy technologies. For example, in the roasting process also molten salts are used to achieve a selective extraction of lithium.^[^ ^31^ ^]^ Other recent technologies, that limit the use of commercial chemicals, involve microwaves.^[^ ^19^, ^32^, ^33^ ^]^ Direct lithium extraction techniques are also proposed.^[^ ^34^ ^]^ Although several methods show potential for efficiently recovering almost all components, their complexity, high costs, and the goal of achieving pollution‐free industrialization have largely confined them to the laboratory scale.^[^ ^8^ ^]^

This highlights the urgent need for a more straightforward, scalable, and industrially feasible approach to recovering spent LIBs.

In this frame, instead of proposing innovative technologies in recovering metals, obtaining limited advantages in comparison to those already proposed, more research attention should be devoted to improving the already available recycling technologies synergies, to propose an integrated approach to whole LIBs recovery, optimizing the efficiency of the resulting process.

### 5.3 Improve Disassembly and Standardization

Literature often highlights the crucial role of eco‐design measures to obtain an optimization of all the components recycling.

Moreover, a major problem consists of a lack of standardization: each manufacturer has a unique design or recipe, adding to the complexity of the process. For example, large components, which are parts of a vehicle, such as the engine can be incorporated in a few minutes, but they require more than 1 h to disassemble.^[^ ^8^ ^]^

It is essential to standardize not only the characteristics of batteries but also the techniques and tools for disassembly, ensuring that authorized treatment centers have adequate information on the composition and disassembly of each part, especially the most valuable components.

### 5.4 Increase Data Transparency and Data Sharing

The discourse surrounding the environmental impacts of lithium mining and recycling is often distorted by the dominance of political and industrial interests. With independent scientific research not being widely shared, public opinion is largely shaped by corporate messaging and media coverage, both of which face limited scrutiny. As a result, critical and alternative perspectives are frequently underrepresented, especially given that many stakeholders—including local communities, NGOs, and state agencies—lack the resources necessary for conducting independent research. This imbalance has led to significant gaps in understanding and diffusing data about the short‐term and long‐term environmental consequences of lithium extraction in various regions.

Ensuring environmental justice requires a transparent approach that addresses the concerns of communities regarding public health, water usage, and waste management before advancing any development projects. However, this effort is complicated by the nascent stage of many emerging technologies and the tendency of companies to closely guard proprietary information about their processes.^[^ ^35^ ^]^

Promote the sharing of research data and findings related to lithium battery recycling through open‐access platforms. This can accelerate the development of new technologies by allowing researchers to build on each other's work without the barriers of proprietary research.

In this frame, the researchers, academics, and journals should guarantee open access to publications, as in the COVID‐19 condition.

**Table** **1** provides a concise overview of proposed strategic actions, highlighting the objective, implementation strategies, and potential challenges for effective lithium supply chain management.

**Table 1:** Table summarizing strategic actions with their objective, implementation strategies, and potential challenges for effective lithium supply chain management.

Strategic action	Objective	Implementation	Barriers
Global accountability in mining monitoring	Establish a unified global system for tracking resource extraction and environmental impacts	– Set up an international platform for reporting and monitoring mining activities, especially in regions with lower environmental and social standards. – Collaborate with governments, NGOs, and local communities to gather comprehensive data on extraction and impacts. – Use remote sensing and real‐time tracking for environmental and social monitoring.	– Limited technological infrastructure in some regions. – Resistance from stakeholders due to proprietary concerns.
Recycling and recovery technologies	Enhance recycling capabilities to reduce dependency on primary lithium sources	– Develop scalable and industrially viable recycling technologies, integrating some methods like pyro‐hydrometallurgy and DLE. – Fund pilot programs for emerging recovery technologies. – Focus research on optimizing current recycling technologies for efficiency and cost‐effectiveness to create a holistic approach to LIB recovery.	– High costs of scaling up technologies. – Limited industrial interest in complex recovery processes. – Regulatory challenges.
Standardization and disassembly optimization	Streamline disassembly and recycling processes through standardized designs	– Create industry‐wide standards for battery composition, labeling, and disassembly. – Require manufacturers to provide documentation on battery composition and disassembly for treatment centers. – Promote eco‐design principles to simplify recycling by reducing material diversity in batteries.	– Manufacturer reluctance to adopt uniform standards. – Complexity of changing existing designs.
Enhancing data transparency and open‐access research	Improve data sharing to foster innovation and public trust	– Develop open‐access platforms for sharing research on lithium extraction, recycling, and environmental impacts. – Ensure transparency in environmental assessments and make ecological impact information publicly accessible. – Facilitate collaborations between academia, research, and industry.	– Corporate resistance due to proprietary concerns. – Limited resources for independent monitoring.

In summary, while the CRMA presents a forward‐looking strategy for securing essential raw materials and fostering economic growth within the EU, it also raises significant questions about environmental sustainability, social impact, and the balance between domestic security and global cooperation. The success of this initiative will largely depend on how these competing interests are managed and whether the EU can truly integrate sustainability into its industrial policies.

## 6 Conclusions

To achieve emission reduction targets and transition away from fossil fuels, the European Union must reevaluate its strategies for energy generation and storage. LIBs are currently the most viable option for powering electric vehicles and providing rapid‐response energy storage, which is essential for maintaining stable energy grids. However, concerns remain about whether there will be enough lithium to meet growing demands and whether this resource can be obtained sustainably. The focus on securing lithium supplies mustn't compromise supply chain justice. The EU must carefully consider the complex ramifications of lithium mining to ensure that the shift to clean energy is both sustainable and equitable.

As global demand for materials continues to rise exponentially, reliance on extraction alone is unsustainable. The prevailing throwaway culture must be replaced with practices that prioritize recycling, reuse, and reduced consumerism. Despite the urgency, society has yet to fully embrace even the first cycle of a truly circular economy. Without substantial and coordinated changes, the outlook remains grim.

To achieve a circular economy in lithium supply, a comprehensive approach is essential, starting with a detailed assessment of lithium resources. This involves identifying and quantifying existing lithium sources, including both primary (mining) and secondary (recycling) resources, while factoring in the environmental and social risks associated with each. Establishing a global database of lithium availability will serve as a foundational reference for sustainable extraction and recycling efforts.

Transparent monitoring across the entire lithium lifecycle, from extraction to recycling, is equally important. Using technologies like blockchain can help trace lithium through the supply chain, ensuring ethical sourcing and environmental responsibility. Creating a centralized system for reporting environmental and social impact data would further support a trustworthy supply chain with minimized risks.

Increasing lithium recovery rates requires expanding recycling infrastructure. This entails building new recycling facilities in key locations and upgrading existing ones with advanced technologies. Both public and private investments are crucial to support this infrastructure expansion, making recycling more accessible and effective. Optimizing current recycling methods is another priority. Research to improve the technologies and standardizing procedures across facilities will lead to higher lithium recovery efficiency, yielding materials ready for reuse in new batteries.

Designing batteries for disassembly is a fundamental step towards easier and more efficient recycling. By encouraging eco‐design and standardization, manufacturers can simplify the disassembly process and material separation. Standards for battery design that allow for simpler disassembly should be enforced, and efforts should be made to use standardized components, reducing complexity in the recycling process and maximizing material recovery.

Collaboration and data sharing among stakeholders in the lithium supply chain are essential to foster innovation and ensure that best practices are widely adopted. Open‐access platforms for sharing data on lithium recycling, recovery rates, and environmental impacts can play a key role, alongside partnerships between industry, government, and academic institutions to accelerate advancements.

A shift in scientific and technological priorities is also necessary. While research often focuses on developing new materials with exceptional properties, there is far less emphasis on separation techniques like demixing, partitioning, decontamination, and recovery of basic elements. These processes, though less glamorous, are critical to the sustainability of new materials and must be prioritized equally. Future research should include realistic collection and recovery rates to accurately assess the availability of recycled materials for manufacturing new batteries. Furthermore, comprehensive approaches, to study the whole recycling system, are essential to give some alternatives and to determine the most promising technologies.

Policy support is vital to drive the adoption of these sustainable practices. Regulatory measures can mandate minimum recycled lithium content in new products, while subsidies for recycling technology investment and tariffs on non‐compliant products can provide strong incentives. A supportive policy environment will promote recycling, and responsible resource management, and encourage companies to adopt sustainable practices.

To mitigate the adverse effects of lithium mining, the EU should adopt strategies that reduce raw material demand and increase governmental oversight within the lithium supply chain. This approach would ensure a sustainable and just energy transition, requiring products to be designed with their entire life cycle in mind,—making them durable, modular, and easy to disassemble—and increase standardization.

## Conflict of Interest

The authors declare no conflict of interest.
